# Fast Fractional Fourier Transform-Aided Novel Graphical Approach for EEG Alcoholism Detection

**DOI:** 10.3390/bioengineering11050464

**Published:** 2024-05-07

**Authors:** Muhammad Tariq Sadiq, Adnan Yousaf, Siuly Siuly, Ahmad Almogren

**Affiliations:** 1 School of Computer Science and Electronic Engineering, University of Essex, Colchester Campus, Colchester CO4 3SQ, UK; m.t.sadiq@essex.ac.uk; 2 Department of Electrical Engineering, Superior University, Lahore 54000, Pakistan; adnan.yousaf@superior.edu.pk; 3 Institute for Sustainable Industries and Liveable Cities, Victoria University, Melbourne 3011, Australia; 4 Department of Computer Science, College of Computer and Information Sciences, King Saud University, Riyadh 11633, Saudi Arabia; ahalmogren@ksu.edu.sa

**Keywords:** electroencephalography, multiscale principal component analysis, alcoholism, ensembled feature selection, neural network classification

## Abstract

Given its detrimental effect on the brain, alcoholism is a severe disorder that can produce a variety of cognitive, emotional, and behavioral issues. Alcoholism is typically diagnosed using the CAGE assessment approach, which has drawbacks such as being lengthy, prone to mistakes, and biased. To overcome these issues, this paper introduces a novel paradigm for identifying alcoholism by employing electroencephalogram (EEG) signals. The proposed framework is divided into various steps. To begin, interference and artifacts in the EEG data are removed using a multiscale principal component analysis procedure. This cleaning procedure contributes to information quality improvement. Second, an innovative graphical technique based on fast fractional Fourier transform coefficients is devised to visualize the chaotic character and complexities of the EEG signals. This elucidates the properties of regular and alcoholic EEG signals. Third, thirty-four graphical features are extracted to interpret the EEG signals’ haphazard behavior and differentiate between regular and alcoholic trends. Fourth, we propose an ensembled feature selection method for obtaining an effective and reliable feature group. Following that, we study many neural network classifiers to choose the optimal classifier for building an efficient framework. The experimental findings show that the suggested method obtains the best classification performance by employing a recurrent neural network (RNN), with 97.5% accuracy, 96.7% sensitivity, and 98.3% specificity for the sixteen selected features. The proposed framework can aid physicians, businesses, and product designers to develop a real-time system.

## 1. Introduction

Alcoholism is a well-known mental illness, recognized for its widespread negative impact and high mortality rates [1]. According to the World Health Organization (WHO), alcohol use contributes to a significant number of deaths, accounting for approximately 5.3 percent (3 million) of total fatalities in 2022 [2]. It ranks as the fifth leading cause of mortality [3] and is identified as the primary risk factor for premature death and disability [4]. Alcoholism profoundly affects overall health, leading to various ailments such as lung and kidney diseases, psychological disorders, and certain types of cancer.

Excessive alcohol consumption contributes to myriad societal issues, including violent crimes, car accidents, social problems, and family breakdowns [5,6]. Individuals with alcoholism often experience mental health challenges, including cognitive impairments, motor difficulties, and prolonged behavioral amendments characterized by restiveness and hopelessness [7,8]. The electrophysiological processes in the brain, including neural signaling and connectivity, undergo significant alterations due to alcohol consumption. These changes manifest as variations in the frequency, amplitude, and connectivity of brainwave patterns. For instance, studies have shown that alcohol intake can enhance certain brainwave frequencies while dampening others [9]. Understanding these complex modifications in brain activity is essential for accurately diagnosing alcoholism and comprehending its neurological implications.

EEG has proven to be a valuable diagnostic and research tool in the field of neuroscience, offering insights into brain dynamics and functioning [10]. The EEG readings reflect the electrical activity generated by neurons in various brain regions. However, interpreting these intricate EEG signals and extracting relevant information can pose challenges. Expert clinicians generally evaluate the signals visually to distinguish between healthy and alcoholic persons. However, even experienced specialists can miss signal fluctuations due to interference [11,12]. Considering the increasing importance of proper neurological abnormality assessment and treatment, this research aims to create an organized investigation structure for efficiently diagnosing alcoholism. A design like this can help with the early detection of probable ailments.

The research literature provides several methods, covering temporal, spectral, non-sequential, auto-regressive (AR), and temporal–spectral strategies, for automatically detecting alcoholic and healthy EEG signals [13]. Both temporal and spectrum analysis methodologies are unsuitable for a thorough evaluation since EEG signals are unpredictable and display fluctuating properties. It is, therefore, essential to use time–frequency analysis techniques. Prior research has evaluated the power distribution of EEG data for classification purposes using AR models and fast Fourier transform (FFT) techniques [14]. To improve the identification process, a study [14] also suggests an automated method incorporating an AR model, a fuzzy-based customizable technique, and a dimension reduction technique.

Numerous studies [15,16] have used a variety of nonlinear attributes to distinguish between normal and alcoholic EEG patterns, including “approximate entropy (ApEn)”, “the largest Lyapunov exponent (LLE)”, “sample entropy (SampEn)”, “correlation dimension (CD)”, “the Hurst exponent (H)”, and “higher-order spectral features”. Recently, “cross-frequency coupling” [17] and “amplitude modulation multiscale entropy” [18] new nonlinear methods have been proposed, offering enhanced capabilities for characterizing the complexity and dynamics of EEG signals. These newer approaches allow for a more comprehensive and detailed analysis of brain states and have the potential to improve the accuracy of alcoholism detection. In addition, time–frequency techniques have been used in studies [19] to separate healthy and alcoholic EEG data.

In recent years, the field of alcoholism detection has witnessed a burgeoning interest in graphical approaches, showcasing the innovative potential of data visualization and computational modeling techniques. Prominent among these methods is the second-order difference plot (SODP), which captures subtle patterns in electroencephalogram (EEG) data by highlighting variations in signal intensity over time. However, the applicability of SODPs is limited by their susceptibility to noise, making them less reliable in real-world, noisy EEG environments [20]. Another approach gaining traction is the analysis of phase-space dynamics, which leverages the concept of attractors to explore the intricate nonlinear dynamics of brain signals. While promising, this method often necessitates the tuning of parameters, making it less user-friendly for non-experts [21]. Graph neural networks (GNNs) have also revolutionized EEG-based alcoholism prediction by modeling neuronal activity as a graph, allowing for the introduction of complicated connections. Nevertheless, for the best results, GNNs may demand significant resources for processing and large datasets, thus limiting their utility in environments with limited resources [22].

Ensemble approaches are emerging as a significant tool for improving the efficacy and robustness of prediction models in the field of EEG-based alcoholism diagnosis. To give an accurate depiction of EEG data, these approaches combine the benefits of numerous feature extraction or classification algorithms. These approaches are widely used in the identification of different neural diseases using EEG but rarely used for alcoholism detection. The model used in [23] integrates input data in tabular, temporal, and picture forms with an ensemble of linear neural networks, long short-term memory (LSTM), and efficient-net classification approaches. This technique, though, has significant disadvantages, including a limiting emphasis on the EEG domain and the use of fundamental engineering traits, high processing costs, and poor classification accuracy. To resolve the aforementioned issues, the key contributions of this study are as follows:

- Improved automated distinction between normal and alcoholic EEG signals through a novel design.
- Developed a noise removal approach tailored for multivariate time-series data.
- Introduced an innovative graphical technique utilizing fast fractional Fourier transform (fast FrFT)) to visualize EEG signals.
- Conducted an evaluation of relevant graphical features for alcoholism detection.
- Implemented an ensemble feature selection strategy to select the most compelling feature set.
- Designed a robust classification model for EEG signal analysis.

The key novelties of this study are the development of a Fast-FrFT-based graphical approach to convert time-series EEGs into topological patterns, graphical feature extraction to understand complex EEG signal behavior, and the proposal of an ensemble feature selection approach for building a realistic alcoholism computerized framework.

## 2. Materials and Methods

EEG recordings from subjects who were both alcohol- and non-alcohol-consuming were used to develop the dataset for this investigation. Anyone can access this dataset for free and academic purposes at https://archive.ics.uci.edu/dataset/121/eeg+database (accessed on 1 January 2023). The recordings come from 64 electrodes positioned on each subject’s head in rest state under the recommended sensor positions established by the American Electroencephalographic Association in 1990. The sampling frequency used for the EEG signals was 256 Hz. The dataset is divided into two parts: standard and alcoholic. The EEG data are divided into 32-second (about 16,400 samples) intervals. The three categories of open-access EEG record files include small sets, large sets, and full records, comprising data from two, ten, and 122 subjects, respectively. This work uses small database collection for research purposes. The dataset details are given in Table 1 [24].

**Figure 1:**
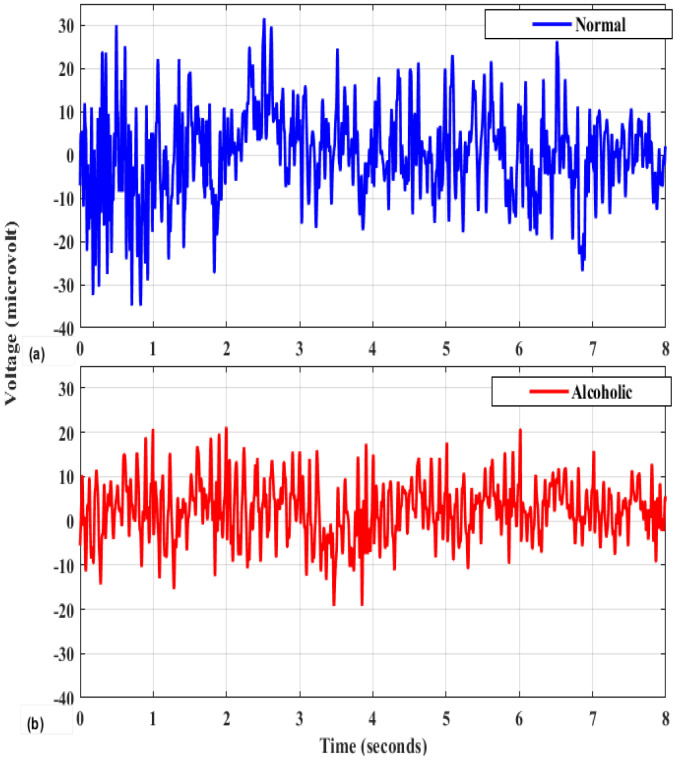
Visualization of EEG signals from individuals with alcoholism and healthy controls. (**a**) normal; (**b**) alcoholic.

Figure 1 presents a graphical depiction of both the alcoholic and control EEG signals.

**Figure 2:**
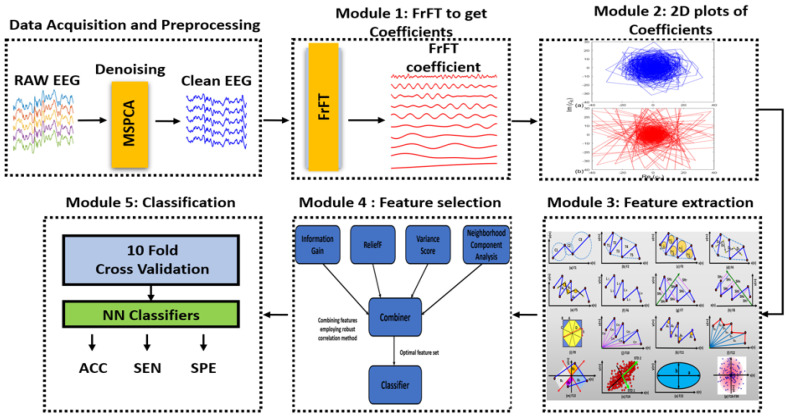
Schematic diagram illustrating the proposed strategy’s components and flow.

This work introduces a graphical technique based on the fast FrFT for identifying normal and alcoholic EEG signals. The comprehensive process of the proposed new framework is segmented into several modules, including preprocessing, a novel graphical technique employing the fractional Fourier transform, feature extraction, ensemble feature selection, and classification. These modules are illustrated in Figure 2. The components mentioned above are discussed further below.

### 2.1. Module 1: Preprocessing

The dataset contains EEG signals captured at a 256 Hz sampling rate and 12-bit resolution for 32 s (about 16,400 samples). The 32 s long EEG recordings are divided into four equal parts, each containing 2048 samples within an 8 s frame. This study uses smaller datasets for research, and the baseline filter efficiently eliminates interference like blinking and physical mobility (>73.3 μV) [25]. The massive electroencephalogram (EEG) data are split into an eight-second frame with four equal parts of 2048 samples for subsequent analysis.

The EEG signals that are recorded from a subject’s scalp are delicate, brittle, and vulnerable to different types of interference, including electrical noise, structured noise, eye movement noise, and others. These interferences have the following mathematical expression:

(1) $$Q=Q_{\mathrm{EEG}}+Q_{N}$$

In the above equation, $Q_{\mathrm{EEG}}$ stands for the information and $Q_{N}$ for the artifact in the signal. The objective is to create a method that efficiently removes noise from the raw signal while maintaining the data in $Q_{\mathrm{EEG}}$. A superior technique for locating correlated data and establishing the direction of the linear relationship between two categories is principal component analysis (PCA). It is crucial to use time–frequency wavelet processing to deal with the EEG signal’s properties because it is nonlinear and not stationary. The use of wavelet decomposition, for this reason, has been tried here. A denoising technique was developed by fusing PCA and the wavelet transform. The algorithm described in [26] is summarized below:

1. Apply the wavelet transform to all channel signals to break them down into their *n* levels.
2. Apply principal component analysis (PCA) on the estimated and detailed matrices of the decomposed signals.
3. Apply the inverse wavelet transform to the resulting principal components (PCs).
4. Consider applying PCA to the equivalent matrix acquired in the previous step to obtain a filtered EEG data sequence. Only a few PCs were preserved in the developed system based on the Kaiser rule, which specifies that PCs with eigenvalues greater than the corresponding individual eigenvalues should be retained. Five layers of wavelets were chosen after multiple testing. To generate the detail and approximation signals, the Sym4 wavelet function was empirically chosen.

**Figure 3:**
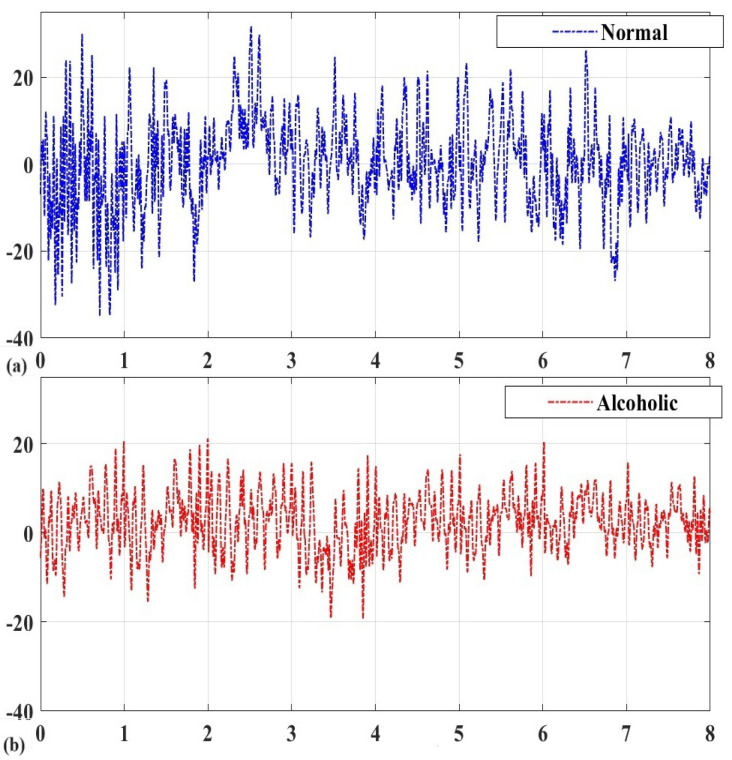
Graphical representation of preprocessing effects: (**a**) normal; (**b**) alcoholic.

Figure 3 presents a graphical representation of the preprocessing effects. It is evident from Figure 3c,d that MSPCA retains the information while effectively removing the noise.

### 2.2. Module 2: Fast Fractional Fourier Transform as Graphical Approach

Fast fractional Fourier transform (fast FrFT) enables the investigation of signals with variable frequencies over time, making it a useful tool for studying non−stationary data like EEG signals [27]. The fast FrFT as a graphical approach may be applied to the analysis of EEG signals to convert time-domain signals into the FrFT domain, producing a 2D representation of the signal in the time–frequency plane. This temporal visual representation of the signal’s frequency content can help in frequency component identification, alcoholism detection, and better interpretation of complex signals. With the fast FrFT’s graphical method, it is possible to identify particular frequency components and their time-varying behavior, leading to more precise neurological condition diagnosis and therapies. The method also offers a more natural way to visualize the signal and spot transitory events that could be challenging to spot when using conventional analysis methods. All things considered, the fast FrFT is a potent tool that can improve the interpretation and comprehension of EEG signals [28]. The fast FrFT, as a graphical approach, enables the identification of specific frequency components and their time-varying behavior, allowing for more accurate diagnoses and treatments of neurological disorders. Additionally, the method provides a more intuitive way of visualizing the signal and detecting transient events that may be difficult to identify using traditional analysis techniques. The fast FrFT is a powerful tool that can enhance the analysis and understanding of EEG signals [28]. As a graphical tool, fast FrFT is described in depth in Algorithm 1.
**Algorithm 1** Fast FrFT Algorithm with Plotting for EEG Signals.Define Parameters:α: The desired fractional Fourier transform angle.*N*: The length of EEG signal x(n).*K*: Scaling factor.Algorithm Steps:1.Input: EEG signal x(n) of length *N* and desired fractional Fourier transform angle α for alcoholism detection.2.Output: EEG signal y(n) in the fractional Fourier domain and a scatter plot of the real and imaginary parts of the fast FrFT coefficients.3.Set the initial value of y(n) to x(n).4.Calculate the scaling factor K=exp(−jπα/2).5.Initialize *k* to 1.6.While $k\leq \log_{2}\left(N\right)$ do:(a)Set FFT length to $2^{k}$.(b)Calculate the filter coefficients $f_{k}\left(m\right)$:
(2)$$f_{k}\left(m\right)=\sqrt{m+\frac{1}{2}}\cdot \exp\left(j\pi K\frac{{\left(m-N/2\right)}^{2}}{2^{k}}\right)$$(c)Filter the EEG signal y(n) using the filter coefficients $f_{k}\left(m\right)$ and perform an FFT to obtain the signal in the fractional Fourier domain.(d)Multiply the signal in the fractional Fourier domain by the scaling factor $K^{2^{k-1}}$.(e)Inverse FFT the signal to obtain the EEG signal in the time domain.(f)Set y(n) to the filtered EEG signal obtained in the previous step.(g)Increment *k* by 1.7.Output EEG signal y(n) in the fractional Fourier domain.8.Calculate the fast FrFT coefficients $c_{k}$:
(3)$$c_{k}=\frac{1}{\sqrt{N}}\sum_{n=0}^{N-1}y\left(n\right)\cdot \exp\left(-j\pi \alpha \frac{{\left(n-N/2\right)}^{2}}{N}\right)\cdot \exp\left(-j2\pi \frac{nk}{N}\right)$$9.Generate a scatter plot of the real and imaginary parts of the fast FrFT coefficients $c_{k}$ for alcoholism detection.

**Figure 4:**
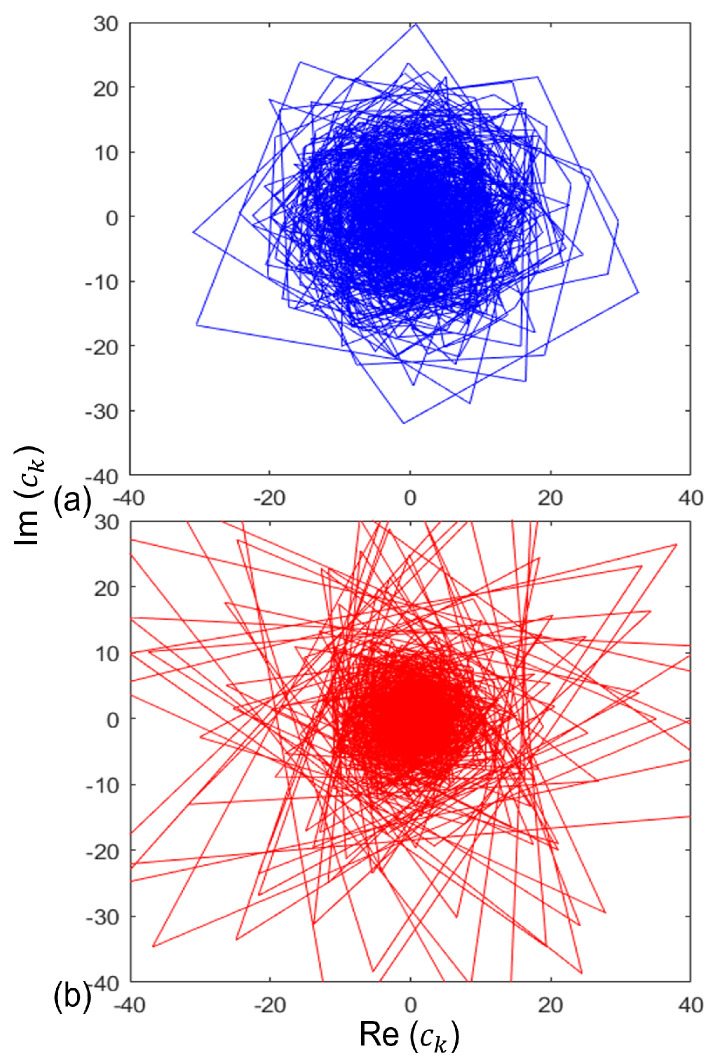
Fractional Fourier transform coefficients for (**a**) alcoholic (blue color) and (**b**) normal (red color) EEG signals. Here, (**a**) 2D shape of normal EEG group covers bigger and wider area, whereas (**b**) 2D shape of alcoholic EEG group is more concentrated towards center.

A 2D representation of fast FrFT is shown in Figure 4.

### 2.3. Module 3: Feature Extraction

Time-domain features, such as mean amplitude, variance, skewness, and kurtosis, define the amplitude and time duration of EEG waves. These traits reveal details regarding the overall structure of the EEG waveform, but they may miss vital frequency-specific facts that can indicate neurological processes. The frequency-domain characteristics describe the power or energy distribution of EEG signals over distinct spectrum bands, such as alpha, beta, theta, and delta. These characteristics can show changes in neurological activity linked with different cognitive or behavioral states and can help recognize frequency-specific behavioral trends in activity.

Graphical features visually depict the EEG signal by combining data from the temporal and frequency domains. These characteristics enable frequency distribution changes over time to be examined and specific trends or factors that may be significant for alcoholic EEG signal categorization to be identified. Graphical aspects have the beneficial feature of providing a more understandable and clear depiction of the EEG signal than numerical values alone. Investigators can find complicated connections and patterns that would be challenging to determine from numerical data alone by visualizing the signal in this manner. It is possible for individuals who consume alcohol to have temporal and spectral changes, and connectivity of brain waves in their EEG signals. For example, alcohol can decrease specific brainwave patterns while enhancing others [6].

**Figure 5:**
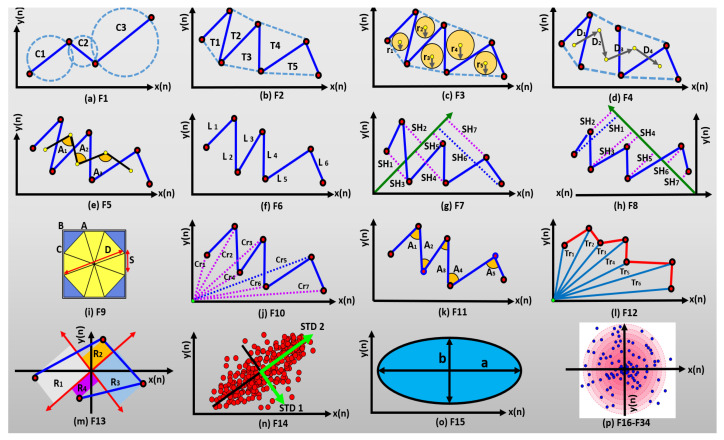
Graphical representations of proposed features.

This study proposes a set of thirty-four novel graphical features, as illustrated in Figure 5, for quantifying and analyzing electroencephalogram (EEG) signals in the context of alcoholism identification. These features capture various aspects of the EEG signal’s characteristics and are designed to provide insights into the variation, complexity, self-similarity, scatter rate, symmetry, and distribution of data points on a 2D space. The details of the thirty-four novel graphical features utilized for the experiments are as follows [21].

F1 (summation of consecutive circles area (SCCA)) measures the variation in the graphical fast FrFT of EEG signals by summing the areas of subsequent circles. F2 (summation of consecutive triangles area (SCTA)) quantifies the 2D dynamics with more flexibility by adding the areas of consecutive triangles. F3 (summation of Heron’s circulars area (SHCA)) captures the self-similarity of the phase-space dynamics through the summation of the areas of Heron’s circulars. F4 (summation of distances between Heron’s circulars (SDHC)) calculates self-sameness and intricacy by adding the distances among succeeding Heron’s circulars. F5 (summation of the angles between Heron’s circulars (SAHC)) quantifies the similarity between 2D shapes using the angles between successive Heron’s circular centers. F6 (summation of successive vector lengths (SSVLs)) captures the amplitude variation in the time domain by summing the lengths of successive vectors. F7 (shortest distance from each point relative to the 45-degree line (SH45)) and F8 (summation of shortest distance from each point relative to the 135-degree line (SH135)) measure the scatter rate of data in different quarters of the 2D shape relative to specific lines. F9 (area of octagon (AOCT)) quantifies the extent of data expansion through the calculation of the area of an octagon. F10 (summation of distances to a coordinate center (SDTC)) measures the variation in the 2D structure from the central point by summing the distances to the center. F11 (summation of angles between three consecutive points (SATP)) captures the smoothness of the 2D shape by summing the angles between three consecutive points.

F12 (summation of triangles areas made by successive points and coordinate center (TACR)) combines the features of SSVLs and SDTC to measure variation and self-similarity simultaneously. It calculates the sum of the areas of triangles formed by connecting successive points to the coordinate center. F13 (summation of consecutive rectangular area (SCRA)) incorporates the features of SH45, SH135, and SDTC to quantify data scattering from multiple reference lines simultaneously. It sums the areas of rectangles formed by the points on specific lines. F14 (two-dimensional standard descriptors (TDSDs)) uses two lines to depict the dispersion of data values on the grid. F15 (elliptical area (ELPA)) captures the elliptical pattern of the EEG signal’s phase-space dynamics by calculating the area of the fitted ellipse. Finally, F16–F34 (central tendency measures (CTMs)) are central tendency measures that provide insights into the distribution of points on the coordinate plane. These measures are calculated for different percentiles and visually represented as circles of varying sizes, reflecting the variability of data points.

Collectively, these thirty-four graphical features offer a comprehensive framework for analyzing EEG signals in the context of alcoholism identification. They provide valuable information about various aspects of the signals’ characteristics and can contribute to more accurate and insightful assessments in this domain.

### 2.4. Module 4: Feature Selection

**Figure 6:**
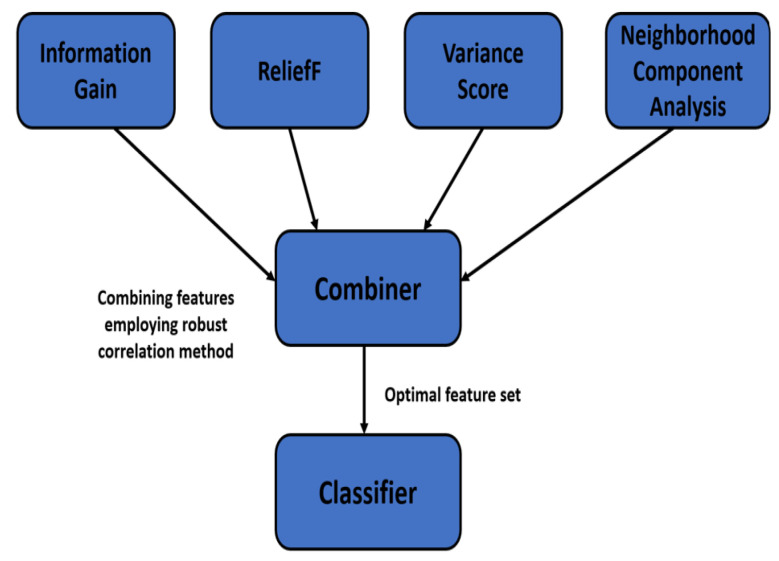
Illustration of ensemble feature selection approach.

Feature selection plays a crucial role in the development of a reliable and accurate algorithm for detecting alcoholism based on EEG signals. The complexity and information contained in EEG signals are immense. Therefore, it is essential to choose pertinent features that accurately depict the symptoms of alcoholism in EEG signals. Feature selection aims to decrease the feature space’s dimensionality while maintaining critical information. As a result, the algorithm becomes more effective and reliable and can precisely identify alcoholism from EEG patterns. We used a novel ensemble feature selection approach in this study. The use of an ensemble feature selection method for EEG feature selection is motivated by the need to circumvent the constraints imposed by individual feature selection methods. Because EEG signals are complex and multidimensional, no feature selection approach can capture all of the vital information from these signals. Ensemble feature selection methods incorporate different feature selection strategies to combine the capabilities of several feature selection methods, thus can overcome the limits of individual methods and give more robust and accurate feature subsets. The proposed ensemble feature selection method is shown in Figure 6 and explained in the subsequent Algorithm 2.
**Algorithm 2** Ensemble Feature Selection.1.Input: A dataset *D* with *m* samples and *n* features, and a positive integer *K* indicating the number of features to select.2.Output: A subset of *K* features that are highly correlated with the target variable but uncorrelated with each other.3.Extract graphical features using fast fractional Fourier transform.4.For i=1 to *n*:(a)Calculate information gain (IG) for feature *i* using the dataset *D* and feature *A*:
IG(D,A)=H(D)−H(D|A)(b)Calculate ReliefF score for feature *i* based on differences between samples:
$ReliefF\left(i\right)=\sum_{j=1}^{m}\frac{-\mathrm{diff}(i,j)}{m}$(c)Calculate variance score for feature *i*:
$Variance\left(i\right)=\frac{1}{m}\sum_{j=1}^{m}{\left(X_{j,i}-{\bar{X}}_{i}\right)}^{2}$(d)Calculate NCA score for feature *i* based on the conditional probability p(i|j):
$NCA\left(i\right)=\sum_{j=1}^{m}p\left(i|j\right)$(e)Calculate CFS score for feature *i* by considering correlations between features and the target variable:
$CFS\left(i\right)=\frac{\mathrm{cor}(X_{i},Y)}{\sqrt{\mathrm{var}\left(X_{i}\right)\cdot \mathrm{var}\left(Y\right)}}\cdot \frac{2}{\mathrm{cor}\left(X_{i},X_{i}\right)+\mathrm{cor}\left(Y,Y\right)}$5.Combine scores for each feature by taking their average or using a weighted average.6.Select the top-K features based on their scores.

### 2.5. Module 5: Classification

The features are then utilized as the input for the classification stage after choosing the optimum feature subset. For EEG alcoholism categorization, several neural network topologies are used in this study. The single-layer neural network (NN), multilayer neural network (MLNN), feed-forward neural network (FFNN), cascade forward neural network (CFNN), recurrent neural network (RNN), and generalized regression neural network (GRNN) are examples of these architectures. Each of these architectures has specific abilities and characteristics that can help to accurately classify EEG data in alcoholism identification.

## 3. Performance Evaluation Parameters

To evaluate the recommended methodology, a 10-fold cross-validation strategy was employed for the classification. This study employs sensitivity, precision, accuracy, F1 score, specificity, and kappa metrics to examine the classification models’ success rate for alcoholism EEG classification tasks. The percent of accurately identified positive instances (alcoholism cases) is measured by sensitivity. In contrast, the fraction of correctly identified positive issues out of the total points projected as positive is represented by precision. The F1 score represents an unbiased assessment of precision and sensitivity, while accuracy reflects the overall accurateness of the model’s outputs. The proportion of accurately diagnosed negative examples (non-alcoholism cases) is measured by specificity. Cohen’s kappa is a statistical measure that assesses the agreement between forecasts and accurate labels while controlling for a chance. These parameters, taken together, provide insights into the ability and reliability of alcoholic EEG classification algorithms, allowing for improved decision making.

## 4. Experimental Setup

The investigations were carried out using a computer system configured with an Intel(R) Core(TM) i7-7500U CPU @2.70 GHz 2.90 GHz processor and 8 GB of memory. The analysis was carried out using MATLAB 2021a and WEKA 3.8.6. Initially, fast FrFT was used to visualize normal and alcoholic EEG signals, yielding 34 graphical features. Each group (normal and alcoholic) had 120 trials, resulting in a 120 × 34 matrix feature vector for every procedure. As a consequence, the feature vector dimensions for the two classes were 240 × 34. An ensemble feature selection method was used to reduce the overall size of the feature vector. Classification results were performed using a 10-fold cross-validation strategy to reduce bias impacts. For 10-fold cross-validation, the feature vector was randomized and separated into ten equal subgroups. The classifier was trained on nine subgroups before being evaluated on the final subset. This procedure was performed ten times to ensure that each subgroup was tested and trained once. The classifier’s effectiveness was assessed by an average of the outcomes of the ten training and testing sessions. Let γ represent the number of features chosen. The training matrix size was 216 × γ, and the test matrix size was 24 × γ for the 10-fold cross-validation.

## 5. Physical Significance of Graphical Features

In this study, 34 graphical features are proposed to capture various aspects of EEG signal characteristics that relate to different neural activities, brain states, and cognitive processes. These features provide a comprehensive framework for quantifying and analyzing EEG signals in the context of alcoholism identification, offering valuable information for both diagnostic and research purposes. The physical significance of the proposed features is summarized in Table 2.

## 6. Statistical Analysis

A statistical analysis was conducted using the Kruskal–Wallis (KW) test to examine the differentiation between normal and alcoholic EEG features. The obtained means, standard deviations (stds), and probability values (*p*-values) for the extracted graphical attributes are presented in Table 3. It is noted that mean values for the normal class are often greater compared to those for the alcoholic class for features 1 to 17, with larger stds observed in the normal class. Conversely, differences in mean and std values between the normal and alcoholic classes for features 18 to 34 suggest potential discriminative capabilities. The *p*-values corresponding to the each features were used to assess the significance of these differences. Based on Table 3, the features with the smallest *p*-values (i.e., *p*-values closest to zero) are considered to have higher discriminative ability between normal and alcoholic EEG signals.

## 7. Results

The results of this section are as follows.

### 7.1. Parameter Selection

We used a comprehensive parameter selection approach to improve classifier performance in EEG alcoholism detection. Various parameter values were investigated for each classifier type, including learning rates, the number of hidden units, activation functions, and epochs. These parameters were chosen based on previous investigations, preliminary testing, and the particular requirements of detecting alcoholism in EEG data. The effectiveness of the classifiers was assessed using standard evaluation measures such as accuracy, precision, recall, F1 score, and specificity, and the values of the parameters were selected by obtaining the highest average score across these indicators. The parameter values establish an appropriate compromise between computational cost and classification performance. The parameter selection approach sought to improve the dependability and accuracy of alcoholism detection outcomes. Based on convergence, accuracy, and the capacity to identify meaningful patterns in the EEG data, ideal parameter values for each classifier, such as learning rates, hidden units, activation functions, and epochs, were discovered. These traits form the basis for further analysis and interpretation of the experimental results. Improved outcomes and higher reliability in EEG alcoholism detection are expected by using these adequately selected parameters. The parameter values of our experiments are tabulated in Table 4.

### 7.2. Alcoholism EEG Signal Detection Results for All and Selected Features

In this section, we present the results of our study on EEG alcoholism detection using a fractional Fourier transform (FrFT)-aided novel graphical approach. Two sets of experiments were conducted: one using all 34 graphical features and the other using selected graphical features obtained through a correlation-based ensemble feature selection method.

**Figure 7:**
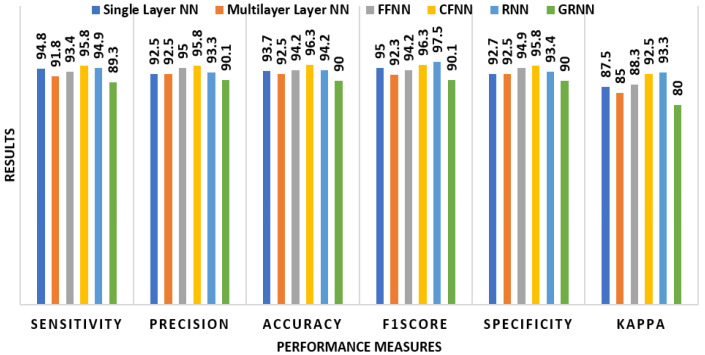
Alcoholism EEG signal detection with all 34 graphical features.

The success rates of several classifiers in alcoholic EEG signal recognition utilizing all 34 graphical features are shown in Figure 7. Across the classifiers, CFNN had the best sensitivity (95.8%) and precision (95.8%), leading to a 96.3% accuracy. RNN had a high F1 score of 97.5% and a specificity of 93.4%. FFNN and single-layer NN also performed well, with accuracy ratings of 94.2% and 93.7%, respectively. GRNN, on the other hand, demonstrated considerably less good metrics for all categories.

**Figure 8:**
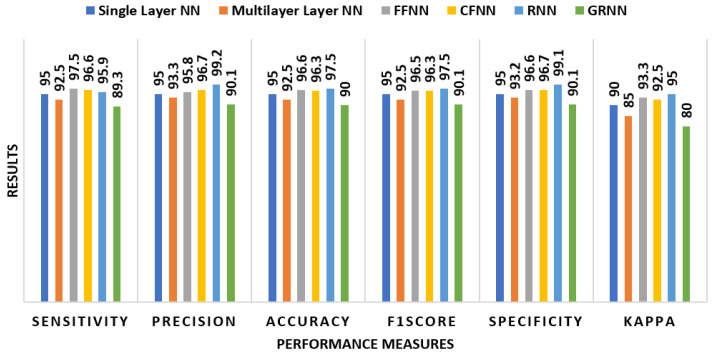
Alcoholism EEG signal detection with selected graphical features obtained with correlation-based ensemble feature selection method.

The findings of alcoholic EEG signal recognition utilizing the chosen graphical features generated by a correlation-based ensemble feature selection approach are presented in Figure 8. The selected graphical attributes (F1, F7, F8, F9, F10, F13, F14, F19, F26, F27, F29, F30, F31, F32, F33, F34) were utilized to evaluate each classifier’s efficacy in detecting alcoholic EEG signals. RNN had the best sensitivity (95.9%) and precision (99.2%), resulting in a 97.5% accuracy. FFNN had a high F1 score of 96.5% and a specificity of 96.6%. CFNN also performed well, with a precision of 96.7% and an accuracy of 96.3%. When examined alongside the results of all 34 graphical features, single-layer NN and multilayer NN exhibited equal performance levels. GRNN, on the other hand, demonstrated consistent results in all measures, as shown in Figure 8.

When the results from Figure 7 and Figure 8 are compared, it is clear that employing the chosen graphical features produced by the correlation-based ensemble feature selection method improves the effectiveness of most classifiers. The selected features determine the critical information relevant to alcoholism EEG signal recognition, leading to improved sensitivity, precision, accuracy, and F1 score for most classifiers. The higher performance of RNN with the selected graphical features implies that the correlation-based ensemble feature selection method proves successful for selecting features that reflect the underlying patterns connected to alcoholism detection. This is due to RNN’s capacity to model temporal dependencies and retrieve useful information from the selected features.

## 8. Discussion

In this study, our objective was to develop a robust graphical technique for distinguishing between normal and alcoholic EEG signals, aiming to achieve improved outcomes. We initiated this process by applying the MSPCA approach to extract clean signals from multivariate EEG data. Subsequently, we delved into the complex and unpredictable behavior of EEG signals using a 2D fractional Fourier transform-aided graphical approach to differentiate between normal and alcoholic classes. Our analysis revealed distinctive characteristics of the 2D shape of EEG signals in the alcoholic group, including a more prominent and wider area, with broader dispersion trends originating from the coordinate center and bisector of trigonometric regions. These findings suggest the potential suitability of this graphical representation as a visual indicator for alcoholism examination in medical practice, facilitating neurologists in comprehending the impact of alcohol on the brain.

A closer look at the mean values among the normal and alcoholic categories, as presented in Table 3, helps in interpreting the observed discrepancies. For instance, feature 1 exhibited a lower mean value in alcoholic subjects, implying a deficiency of this feature in alcoholics compared to non-alcoholics. In contrast, feature 11 displayed a higher mean value in alcoholic subjects, indicating a higher occurrence of this feature in alcoholics. These distinctions provide valuable insights into the dataset’s properties and trends, contributing to a clearer understanding of the association between graphical features and alcoholism. An effective feature selection approach is essential to effectively comprehend the structural characteristics of normal and alcoholic EEG signals. In response to the challenges posed by various feature selection methods, we proposed a novel correlation-based ensemble feature selection method for EEGs.

Table 5 provides a comprehensive analysis of our proposed computerized approach for alcoholism detection using EEG signals in comparison with existing studies, including [24,29,30,31,32,33,34,35], which predominantly employ wavelet analysis. While wavelet techniques have been widely utilized in EEG signal processing, it is essential to acknowledge their inherent limitations, including the trade-off between time and frequency resolution, subjectivity in parameter selection, challenges in interpretability, and the assumption of signal stationarity. In light of these considerations, the proposed method offers several advantages, as described below.

**Parameter independence:** The primary benefit of our suggested method is that it does not rely on any specific parameter adjustments. The fast FrFT technique is based on a desirable fractional Fourier transform angle α, which can be selected based on the unique analytic objectives. This parameter independence removes the requirement for parameter fine-tuning or optimization, making the procedure simpler for users and less susceptible to subjective bias.

**Time–frequency localization:** The fast FrFT approach uses the time–frequency plane representation produced from EEG signal processing. This approach allows a more localized view of frequency components over time, allowing more accurate analysis of non-stationary signals. By visualizing the signal in the time–frequency plane, our suggested method can aid in understanding and detecting transitory occurrences or patterns by facilitating the recognition of specific frequency components and their time-varying behavior.

**Graphical representation:** A scatter plot of the real and imaginary sections of the fast FrFT coefficients is generated using the fast FrFT method. This graphical representation enables easy visualization and investigation of the signal’s properties. Visually identifying and examining patterns, anomalies, or specific features provides vital insights into the fundamental structure of the EEG data. This graphical approach can be beneficial for spotting complex patterns or minor alterations that would be difficult to discover using other techniques.

Table 5 includes details about the method and features used, feature selection approaches, cross-validation methods, classifiers, and accuracy, sensitivity, and specificity scores. Our proposed work uses the FHWT with matrix determinant as features for detecting alcoholism, without employing feature selection. The classifier used is RNN, and a 10-fold cross-validation approach is applied. Our proposed work achieves an accuracy of 93.3%, with equal sensitivity and specificity. Comparing our work with existing studies, it is evident that various methods, features, feature selection strategies, cross-validation methods, and classifiers have been employed in the literature. While our proposed work achieves an accuracy of 93.3%, some previous research reports higher accuracy values, such as 97.91%, 98.91%, and 99.16%. It is important to note that our work does not employ feature selection, which might explain the slightly lower accuracy compared to certain previous studies.

Furthermore, our research includes two variations: one that utilizes all features obtained from FHWT and employs the CFNN classifier, resulting in an accuracy of 96.3% and sensitivity and specificity of 95.8%. The alternative variation uses the CFS technique to select specific features and employs the RNN classifier, achieving an accuracy of 97.5%, a sensitivity of 96.7%, and a specificity of 98.3%. In summary, our proposed computerized study demonstrates competitive accuracy in alcoholism detection using EEG signals.

**Figure 9:**
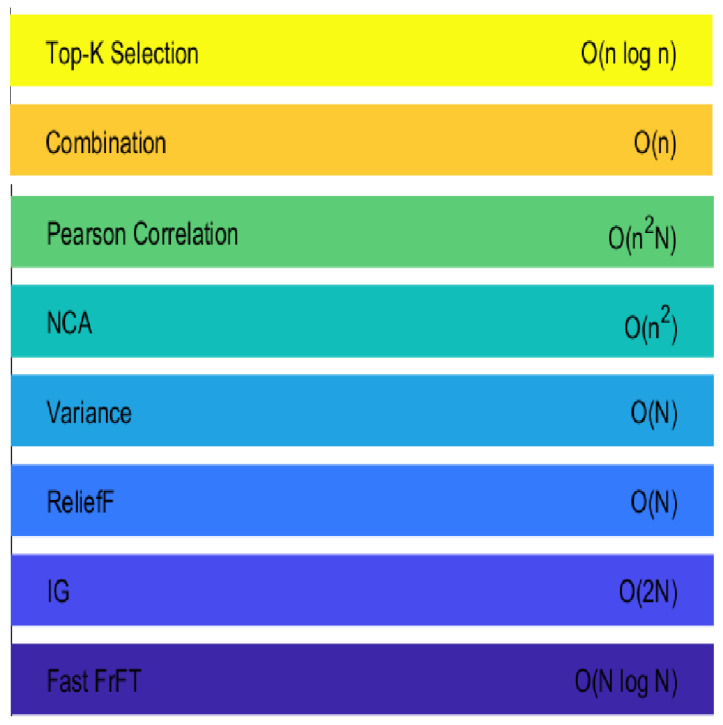
Computational costs of feature selection algorithm.

It is also important to consider the computational expense of our suggested method, as indicated in Figure 9. The complexity varies for each step, with the fast FrFT step having a computational complexity of O(NlogN), where *N* represents the length of the EEG signal. The Pearson correlation step involves a complexity of $O(n^{2}N)$ when evaluating correlations among features and the target variable and among pairs of features, with a combined step complexity of O(n). The top-*K* selection step has a complexity of O(nlogn). Importantly, our method offers parameter independence, removing the need for parameter fine-tuning or optimization, making it user-friendly and less susceptible to bias. It also provides improved time–frequency localization and graphical representation, which facilitates the recognition of specific frequency components and time-varying behaviors in EEG data.

**Figure 10:**
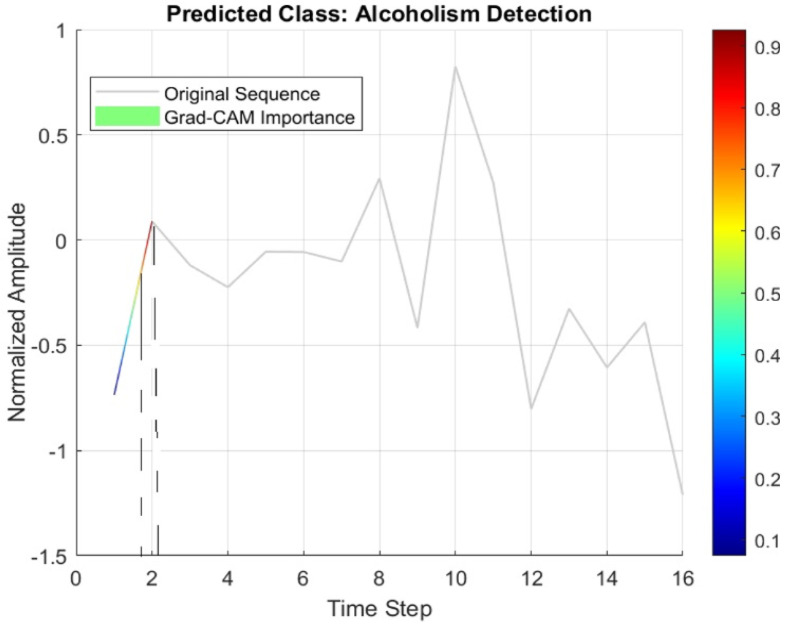
Grad−CAM for visualization of model’s decisions.

Figure 10 presents a combined plot overlaying the original sequence with gradient-weighted class activation mapping (Grad-CAM). Grad-CAM helps to visualize the time steps in the input sequence that were crucial for the alcoholism identification, as shown by dashed horizontal lines in Figure 10. This approach not only aids in identifying influential time steps relied upon by the model but also facilitates the detection of potential data irregularities, ensuring the reliability and robustness of the analysis.

While our study presents promising results, it is essential to acknowledge some limitations and consider future research directions:

1. Data size: The study’s efficacy can be further evaluated with larger datasets to enhance the model’s robustness and generalizability.
2. Clinical validation: Collaborating with medical professionals for clinical validation and testing on real-world EEG datasets is essential.
3. Model optimization: Further optimization of model parameters and architecture might enhance the accuracy and efficiency.
4. Incorporation of explainable machine learning: Understanding the importance of explainable artificial intelligence, particularly Shapley additive explanations; we plan to include these methods in our future work. This addition will contribute to the interpretability of our models, providing valuable insights into the features and patterns influencing the predictions.

By addressing these limitations and pursuing future research in these directions, we aim to provide a more comprehensive and effective solution for EEG-based alcoholism detection.

## 9. Conclusions

In conclusion, this study proposes a novel paradigm for detecting alcoholism using EEG signals. The proposed system entails eliminating interference and artifacts from EEG data, visualizing the chaotic aspects of the signals, extracting graphical features to distinguish between normal and alcoholic trends, and using a novel ensemble feature selection method. The experimental findings show that the proposed strategy provides up to a 14.98% classification accuracy improvement when employing an RNN. The suggested framework has the ability to aid in the detection of alcoholism in real time, which will benefit physicians, businesses, and product designers. The proposed fast FrFT method offers advantages such as parameter independence, time-frequency localization, and a graphical representation of the signal. These characteristics make it a promising approach for analyzing non-stationary EEG signals.

## Figures and Tables

**Table 1:** Alcoholism EEG dataset details. Abbreviations: During data collection, two types of stimuli were utilized: Single (s1) and dual stimuli (s1 matching s2 and s1 not matching s2).

Subjects	Stimuli Present	No. of Segments	Sampled Frequency	No. of Samples
	s1 obj			
Normal	s2 match	120	256	2048
	s2 no match			
	s1 obj			
Alcoholic	s2 match	120	256	2048
	s2 no match			

**Table 2:** Physiological information provided by EEG features.

Feature	Physiological Information
F1 (SCCA)	Reflects changes in cyclic patterns and oscillations in EEG signals; captures variations in power of specific frequency bands like alpha and beta waves.
F2 (SCTA)	Quantifies 2D dynamics of EEG signals; represents variations in signal complexity, indicating more dynamic brain activity or cognitive processes.
F3 (SHCA)	Captures self-similarity in EEG phase-space dynamics, occurring during different brain states such as sleep cycles or meditation, where specific patterns repeat.
F4 (SDHC)	Reflects self-sameness and intricacy in EEG signal patterns, indicating the degree of regularity and intricacy that varies based on cognitive tasks or mental states.
F5 (SAHC)	Quantifies similarity between different segments of EEG signals; provides information about coherence and consistency of neural activity relevant to various brain processes.
F6 (SSVLs)	Captures variations in EEG signal amplitudes; changes associated with different levels of neural activity, such as sensory input intensity or sleep depth.
F7 (SH45) and F8 (SH135)	Measure scatter rate of data in different quarters of the 2D shape relative to specific lines; high scatter rates indicate diverse neural activities or cognitive states.
F9 (AOCT)	Quantifies extent of data expansion in EEG signals; reflects spatial coverage of neural activity across the scalp.
F10 (SDTC)	Measures variation in EEG signal structure from the central point; provides insights into distribution of EEG signal features related to focal or diffuse neural activities.
F11 (SATP)	Captures smoothness of 2D shape formed by EEG signal data points; indicative of regular and consistent neural activity.
F12 (TACR)	Combines variations and self-similarity, indicating variations in EEG signal patterns while considering the self-similarity of the dynamics.
F13 (SCRA)	Quantifies data scattering from multiple reference lines simultaneously; captures complex EEG signal patterns and their distribution in various directions.
F14 (TDSDs)	Offers insights into distribution patterns of EEG data values in a 2D space; reveals patterns of neural activity and their variability.
F15 (ELPA)	Captures elliptical patterns in EEG signals’ phase-space dynamics; patterns may relate to specific neural processes exhibiting elliptical trajectories.
F16–F34 (CTMs)	Central tendency measures offering insights into distribution of EEG signal data points; quantify how EEG data values cluster around central tendencies, reflecting regularity and variability in neural activity.

**Table 3:** Means, standard deviations, and *p*-values of extracted graphical features.

Feature	Mean (Normal)	Std (Normal)	Mean (Alcoholic)	Std (Alcoholic)	*p*-Value
1	−3221.4	24,370	−4016.8	10,657	0.023
2	80,296	31,078	40,113	20,512	0.001
3	2655.8	1784.9	3307.2	2442.3	0.005
4	26,212	3787.7	16,754	3683.5	0.002
5	$1.47\times 10^{5}$	7209.3	$1.46\times 10^{5}$	8869	0.004
6	−1025.4	7757.2	−1278.6	3392.3	0.006
7	14,196	2107.6	8760.6	1933	0.003
8	16,905	2558.1	10,033	2329.7	0.001
9	4188.8	4808.5	1367.4	1319	0.008
10	11,653	1728.5	7219.8	1595.4	0.007
11	$1.62\times 10^{5}$	5741.4	$1.64\times 10^{5}$	6167.8	0.002
12	8406.4	729.25	6844.1	850.14	0.009
13	$1.86\times 10^{5}$	53,453	64,162	29,440	0.005
14	364.47	103.62	123.97	55.761	0.003
15	$1.29\times 10^{6}$	$5.70\times 10^{6}$	$1.14\times 10^{5}$	$3.05\times 10^{5}$	0.006
16	0.9722	6.9677	−0.21973	5.357	0.008
17	0.72619	11.567	−0.098082	9.4814	0.004
18	−0.47366	14.808	1.6558	12.05	0.001
19	−4.8039	18.333	1.464	13.363	0.002
20	−1.7399	22.051	−3.5912	17.59	0.003
21	−1.0148	32.037	−1.713	21.282	0.004
22	−0.67079	39.375	−0.70012	27.492	0.005
23	1.7148	57.429	4.2366	32.64	0.006
24	−5.5346	84.909	6.281	35.177	0.007
25	0.5096	111.75	5.887	46.402	0.008
26	9.1545	147.14	4.8485	61.202	0.009
27	11.607	185.77	0.64924	76.274	0.01
28	14.254	235.02	−2.7439	87.751	0.011
29	62.792	298.06	6.8594	109.18	0.012
30	−28.614	382.41	0.52544	152.05	0.013
31	21.068	483.6	4.1776	170.73	0.014
32	−11.236	657.59	−2.8417	235.52	0.015
33	−49.316	861.98	10.273	310.28	0.016
34	191.59	1399.6	−47.655	503.53	0.017

**Table 4:** Parameter selection for EEG alcoholism detection.

Classifier	Parameter 1	Parameter 2	Parameter 3	Parameter 4
Single-Layer NN	Learning Rate: 0.01	Hidden Units: 50	Activation: ReLU	Epochs: 100
Multilayer NN	Learning Rate: 0.05	Hidden Units: 100	Activation: Tanh	Epochs: 200
FFNN	Learning Rate: 0.1	Hidden Units: 200	Activation: Sigmoid	Epochs: 150
CFNN	Learning Rate: 0.01	Hidden Units: 100	Activation: ReLU	Epochs: 100
RNN	Learning Rate: 0.01	Hidden Units: 50	Activation: Tanh	Epochs: 200
GRNN	Sigma: 0.5	Radius: 0.1	Activation: Gaussian	-

**Table 5:** Comparison of the proposed computerized work with available work. Abbreviations: FS—feature selection, CV—cross-validation, Acc—accuracy, Sen—sensitivity, Spe— specificity.

Method, Feature (Ref.)	FS	CV	Classifier	Acc	Sen	Spe
EWT+Statistical Features (Anuragi et al., 2020 [24])	*p*-value	Leave one out	LS-SVM	98.75	98.35	99.16
HMMs+Coupled HMMs (Zhong et al., 2002 [36])	Not used	10-fold	NN	82.98	-	-
Nonlinear+HOS Features (Acharya et al., 2012 [37])	*p*-value	3-fold	SVM	91.7	90	93.33
WPT+HOS Features (Faust et al., 2013 [29])	*p*-value	10-fold	KNN	95.8	95.8	95.8
CWT+Statistical Features (Upadhyay et al., 2014 [30])	Not used	10-fold	SVM	94.29	-	-
TQWT+Nonlinear Features (Patidar et al., 2017 [31])	PCA	10-fold	LS-SVM	97.02	96.53	97.5
Granger Causality (Bae et al., 2017 [38])	Not used	5-fold	SVM	90	95.3	82.4
DTCWT+Nonlinear Features (Sharma et al., 2018 [32])	*p*-value	10-fold	SVM	97.91	-	-
TBOWFB+Nonlinear Features (Sharma et al., 2018 [33])	*p*-value	10-fold	LS-SVM	97.08	97.08	97.08
Synchronization Likelihood (Mumtaz et al., 2018 [39])	ROC	10-fold	SVM	98	99.9	95
EMD+Power Band+Fractal Dimension (Thilagaraj et al., 2019 [34])	ICA	10-fold	KNN	98.91	99.02	99.24
FBSE-EWT+Nonlinear Features (Anuragi et al., 2020 [35])	Not used	Leave one out	LS-SVM	98.8	98.3	99.1
NN (Siuly et al., 2020 [40])	Not used	10-fold	LSTM	93	95	92
FHWT+Matrix Determinant (This work)	No	10-fold	RNN	93.3	93.3	93.3
FHWT+All Features (This work)	No	10-fold	CFNN	96.3	95.8	95.8
FHWT+Selected Features (This work)	CFS	10-fold	RNN	97.5	96.7	98.3

## Data Availability

The EEG database used in this study is publicly available on the UCI Machine Learning Repository at https://archive.ics.uci.edu/dataset/121/eeg+database.
